# Abundant *NDRG2* Expression Is Associated with Aggressiveness and Unfavorable Patients’ Outcome in Basal-Like Breast Cancer

**DOI:** 10.1371/journal.pone.0159073

**Published:** 2016-07-11

**Authors:** Vera Kloten, Martin Schlensog, Julian Eschenbruch, Janina Gasthaus, Janina Tiedemann, Jolein Mijnes, Timon Heide, Till Braunschweig, Ruth Knüchel, Edgar Dahl

**Affiliations:** Molecular Oncology Group, Institute of Pathology, Medical Faculty of the RWTH Aachen University, Aachen, Germany; University of Navarra, SPAIN

## Abstract

*NDRG2*, a member of the N-myc downstream-regulated gene family, is thought to be a putative tumor suppressor gene with promising clinical impact in breast cancer. Since breast cancer comprises heterogeneous intrinsic subtypes with distinct clinical outcomes we investigated the pivotal role of NDRG2 in basal-type breast cancers. Based on subtype classified tumor (n = 45) and adjacent normal tissues (n = 17) we examined *NDRG2* mRNA expression and CpG-hypermethylation, whose significance was further validated by independent data sets from The Cancer Genome Atlas (TCGA). In addition, NDRG2 protein expression was evaluated immunohistochemically using a tissue micro array (TMA, n = 211). *In vitro*, we investigated phenotypic effects caused by NDRG2 silencing in the basal A-like HCC1806 as well as NDRG2 over-expression in basal A-like BT20 compared to luminal-type MCF7 breast cancer cells. Our tissue collections demonstrated an overall low *NDRG2* mRNA expression in breast cancer subtypes compared to normal breast tissue in line with an increased CpG-hypermethylation in breast cancer tissue. Independent TCGA data sets verified a significant (P<0.001) expression loss of *NDRG2* in breast tumors. Of interest, basal-like tumors more frequently retained abundant *NDRG2* expression concordant with a lower CpG-hypermethylation. Unexpectedly, basal-like breast cancer revealed an association of *NDRG2* expression with unfavorable patients’ outcome. In line with this observation, *in vitro* experiments demonstrated reduced proliferation and migration rates (~20%) in HCC1806 cells following *NDRG2* silencing. In contrast, NDRG2 over-expressing luminal-type MCF7 cells demonstrated a 26% decreased proliferation rate. Until now, this is the first study investigating the putative role of NDRG2 in depth in basal-type breast cancer. Our data indicate that the described putative tumor suppressive function of NDRG2 may be confined to luminal- and basal B-type breast cancers.

## Introduction

Breast cancer remains the most frequently diagnosed cancer and the leading cause of cancer deaths in European women [1]. Based on the high breast cancer-related mortality rate, the understanding of tumor biological and molecular consequences is mandatory, enabling an individual and targeted development of breast cancer therapy. However, adapting current diagnostic and therapeutic strategies to each patient is a challenging task due to the heterogeneous molecular aspects of breast tumors. Breast cancer can be classified into four main intrinsic subtypes, i.e. luminal A, luminal B, HER2-enriched and basal-like, based upon global gene expression profiles demonstrated for the first time in 2000 by Perou and colleagues [2]. While particularly patients with luminal A tumors benefit from systemic endocrine therapy, therapeutic targets for the basal-like class of breast cancer is still insufficient due to the lack of understanding of the driving oncogenic mechanisms [3], resulting in chemotherapy treatment. In addition, breast cancer patients affected with basal-type cancer show worse survival outcome compared to patients with e.g. luminal A-type breast cancer [3].

A putative tumor suppressor gene implicated in cancer development [4] and progression [5–7] is NDRG2, a member of the N-myc downstream-regulated gene family. NDRG2 has been widely implicated in carcinogenesis including breast cancer invasion [8], angiogenesis [9] and metastasis [10–12]. Recent studies indicated decreased *NDRG2* expression due to promoter DNA-hypermethylation [13,14], underlining a possible tumor suppressive role of NDRG2 in breast carcinogenesis. So far, NDRG2 was shown to inhibit invasive and metastatic capacity of breast cancer cells by reducing the production of active TGF-β [12] or suppression of MMP-9 activity [15]. Recently, Kim et al. [11] demonstrated a retarded STAT3 signaling by NDRG2 resulting in an inhibition of EMT progression due to the down-regulation of SNAIL expression. Nevertheless, studies evaluating the putative tumor suppressive biological and clinical impact of NDRG2 were irrespective of intrinsic breast cancer subtypes or mainly focused on luminal or basal B breast cancer cell models *in vitro* and *in vivo*.

This is the first study giving evidence that the described putative tumor suppressive function of NDRG2 may be confined to luminal- or basal B-type breast tumors: A more frequently retained *NDRG2* mRNA expression associated with unfavorable clinical outcome in basal-type breast cancer patients. Moreover, NDRG2 knockdown in the basal A breast cancer cell line HCC1806 caused a reduced proliferation and migration rate while NDRG2 over-expression in basal A-like BT20 breast cancer cells, lacking endogenous NDRG2 expression, resulted in an increased cell proliferation. In contrast, over-expression of NDRG2in luminal MCF7 cells showed a 26% decreased proliferation rate compared to control cells while significance slightly missed (P = 0.064).

## Methods

### TCGA patients’ data set and breast cancer-related online tools

Data from breast cancer, normal and metastatic tissue specimen were used from The Cancer Genome Atlas (TCGA) [16], comprising overall patients' data of two independent platforms: Illumina Infinium DNA methylation chip (HumanMethylation 450K array) and gene expression IlluminaHiSeq (n = 999 patients). The data of this study can be explored using the cBioPortal for Cancer Genomics (http://cbioportal.org). An overview of the clinical characteristics of breast cancer patients is summarized in S1 Table. An independent univariate survival analysis of overall survival (OS) and relapse-free survival (RFS) was analyzed based on a merged data set (n = 4,142 breast cancer samples) from the Kaplan Meier-Plotter [17].

### Subtype-specific patients’ tissue collective

A total of 62 tissue samples, including breast tumors (n = 45) and adjacent normal tissues (n = 17) were obtained through the RWTH centralized biomaterial bank (RWTH cBMB). All patients gave written informed consent for retention and analysis of their tissue for research purposes (local ethical review board of the medical faculty of the RWTH Aachen, ref no. EK-206/09). Tumor material was snap-frozen in liquid nitrogen directly after surgery. Haematoxylin and eosin-stained sections were prepared for assessment of the percentage of tumor cells. The median percentage of vital tumor cells was 100% in the selected samples. Breast cancer molecular subtypes were defined according to St. Gallen criteria [18]. Ki67 staining was performed to make a distinction between luminal A and luminal B breast tumors. The Ki67 staining was performed at the Uniklinik RWTH Aachen pathology department, according to standard protocols. The percentage of cells stained positive for Ki67 was determined using the free available ImmunoRatio software [19]. An overview of the clinical characteristics of breast cancer patients of this study is summarized in S2 Table.

### Cell lines

Basal A-type HCC1806 and BT20 as well as luminal-type MCF7 breast cancer cell lines were obtained from the American Type Culture Collection (ATCC, Manassas, VA), which assures the molecular authentication of cell lines (ATCC Bulletin 2010: *Maintaining high standards in cell culture*, https://www.atcc.org/~/media/PDFs/CellBiologyStandards.ashx). MCF7, HCC1806 and BT20 cells were cultured in RPMI media (Gibco Life Science) supplemented with 10% fetal calf cerum (FCS), 1% L-Glutamin and penicillin/streptomycin. MCF7 cell medium additionally complemented with 1% sodium pyruvat, 1% non-essential amino acids and 1% insulin. All cell lines were incubated in a humidified incubator at 37°C supplied with 5% carbon dioxide. Cells were regularly tested for Mycoplasma infection using the PCR-based Venor® GeM Mycoplasma Detection Kit (Minerva Biolabs, Berlin, Germany).

### Nucleic acid extraction and reverse transcription PCR

Total cellular RNA from breast cancer and normal breast tissues as well as cultured breast cancer cells was prepared by using TRIzol reagent (Invitrogen-Life Technologies). cDNA was synthesized using the reverse transcription system (Promega, Madison, WI) as previously described [20].

### Real-time PCR

cDNAs were amplified by semi-quantitative real-time PCR using SYBR-Green PCR mix (Bio-Rad Laboratories, Munich, Germany) performed in an iCycler IQ5 (Bio-Rad Laboratories) and quantified as previously described [21]. All used primers spanned at least one intron, and are listed in S3 Table.

### Pyrosequencing

Pyrosequencing analysis of a distinct *NDRG2* promoter CpG-region was performed by using the PyroMark PCR Kit (Qiagen) for initial fragment amplification. Afterwards, the PyroMark96 ID device and the PyroGoldSQA Reagent Kit (Qiagen) were implemented as previously described [22]. The *NDRG2* assay was designed by using the PyroMark Assay Design Software (Qiagen). Used *NDRG2* pyrosequencing primers are listed in S3 Table.

### Bisulfite modification

The extracted tissue DNA was bisulfite-converted using the EZ DNA methylation kit (Zymo Research, Orange, CA) as previously described [20].

### Western blot analysis

For Western blot analysis, cultured cells were washed in ice-cold PBS solution and prepared under reducing (50 mM DTT) conditions by using NuPAGE LDS electrophoresis sample buffer (Invitrogen-Life Technologies). Samples were separated on a 4–12% polyacrylamide gel (Invitrogen-Life Technologies) using MOPS-SDS running buffer. Proteins were electroblotted to nitrocellulose membranes and unspecific binding sites were blocked in TBS-T [10 mM Tris-HCl, 150 mM NaCl, 0.1% (v/v) Tween 20, pH 7.6] containing 5% (w/v) non-fat milk powder. The membranes were then probed overnight with a polyclonal rabbit anti-NDRG2 (Atlas Antibodies, HPA002896, 1:500) (4°C). Membranes were washed three times with TBS-T and incubated with horseradish peroxidase-conjugated secondary antibodies (Dako, Glostrup, Denmark), and the signal was detected by chemiluminescence (Pierce ECL, Thermo Scientific, Rockford, IL). Equal protein loading was monitored by probing with a ß-actin specific antibody (Sigma-Aldrich (A5316), 1:2000).

### Immunohistochemistry

NDRG2 protein expression was assessed using a TMA with 161 breast cancer and 50 normal tissue cases that have been described previously [20,23]. Paraffin-embedded tissue sections (2 μm) were subjected to immunostaining using the UltraVision Quanto Detection System HRP (Thermo Scientific) following the manufacturer’s instructions. Antigen retrieval was performed by pre-treatment in citrate buffer (pH 6) in a microwave oven (30 min). The sections were incubated for 1 h at room temperature with anti-NDRG2 (1:150). Slides were incubated for 10 min with secondary antibody (HRP Polymer Quanto; Thermo Scientific). DAB Quanto chromogensubstrate (Thermo Scientific) was used for antibody detection. An experienced breast cancer pathologist scored the immunohistochemical staining intensity according to the scoring system suggested by Remmele and Stegner (1987).

### Transient transfection

Transient transfection of human BT20 and MCF7 breast cancer cells with NDRG2-pT-Rex-DEST 30 (Invitrogen-Life Technologies) expression vector, containing the full-length human *NDRG2* cDNA, was performed as recently described [20]. In brief, 6 x 10^5^ cells per 6-well were transfected using the FugeneHD reagent (Invitrogen-Life Technologies) at a ratio of 2:6 (DNA:reagent) for 48 h. Thereafter cells were cultured for one week in complete medium under positive selection using geneticin (G418, 100 μg/ml).

### RNA Interference

Human HCC1806 breast cancer cells (6 x 10^5^) were transfected with HiPerfect transfection reagent (Qiagen) applying two predesigned siRNA directed against human NDRG2 (Hs_NDRG2_4 FlexiTube siRNA, Cat. no. SI00656096, 5’-AAGGGTATGGACCTACGTGAA-3’ and Hs_NDRG2_6 FlexiTube siRNA, Cat. no. SI04222666, 5’-CTCGCCTGTCCCGGTCTCGTA-3’ (20 nM each)) and a combination of both according to the manufacturer’s instructions (Qiagen). After 48 h treatment cells were splitted and re-transfected with siRNA to guarantee efficient NDRG2 knock-down. In addition, a commercial non-silencing control siRNA (5’-AATGCTGACTCAAAGCTCTG-3’) (Qiagen) served as negative control. After 48 h, 96 h, and 144 h, samples were harvested for total RNA and protein isolation.

### Cell proliferation assay

The XTT proliferation assay (Roche) for BT20 and MCF7 (gain-of-function) as well as HCC1806 (loss-of-function) cell models was used and performed as previously described [20].

### Cell growth assay

Increase of cell number was recorded for BT20 and MCF7 NDRG2-positive and negative cells over 96 h. 2 x 10^4^ cells were seeded in 6-well culture plates and cell number was determined with the *CASY® Cell Counter and Analyzer* (OLS OMNI Life Science, Bremen, Germany) after 24 h, 48 h, 72 h and 96 h. Experiments were performed in triplicate.

### Wound healing (“Scratch”) assay

The *in vitro* motility was assessed by performing a monolayer scratch wound assay in BT20 and HCC1806 cell models as previously described [24].

### Statistical analysis

Statistical analyses were performed using SPSS 22.0 (SPSS, Chicago, IL) and GraphPad Prism 5.0 (GraphPad Software Inc., La Jolla, CA). Box Plot graphs are shown as follows: *Horizontal lines*: grouped medians. *Boxes*: 25–75% quartiles. *Vertical lines*: range, peak and minimum. The non-parametric Mann-Whitney U-test was used in order to compare *in vitro* results of the control and NDRG2 set, respectively. Correlation analysis was performed by calculating a *Spearman* correlation coefficient. Differences were considered statistically significant if the two sided p-values were equal or below 5% (≤0.05).

## Results

### *NDRG2* revealed a divergent expression and methylation pattern in basal- compared to luminal-type breast cancer

**Fig 1:**
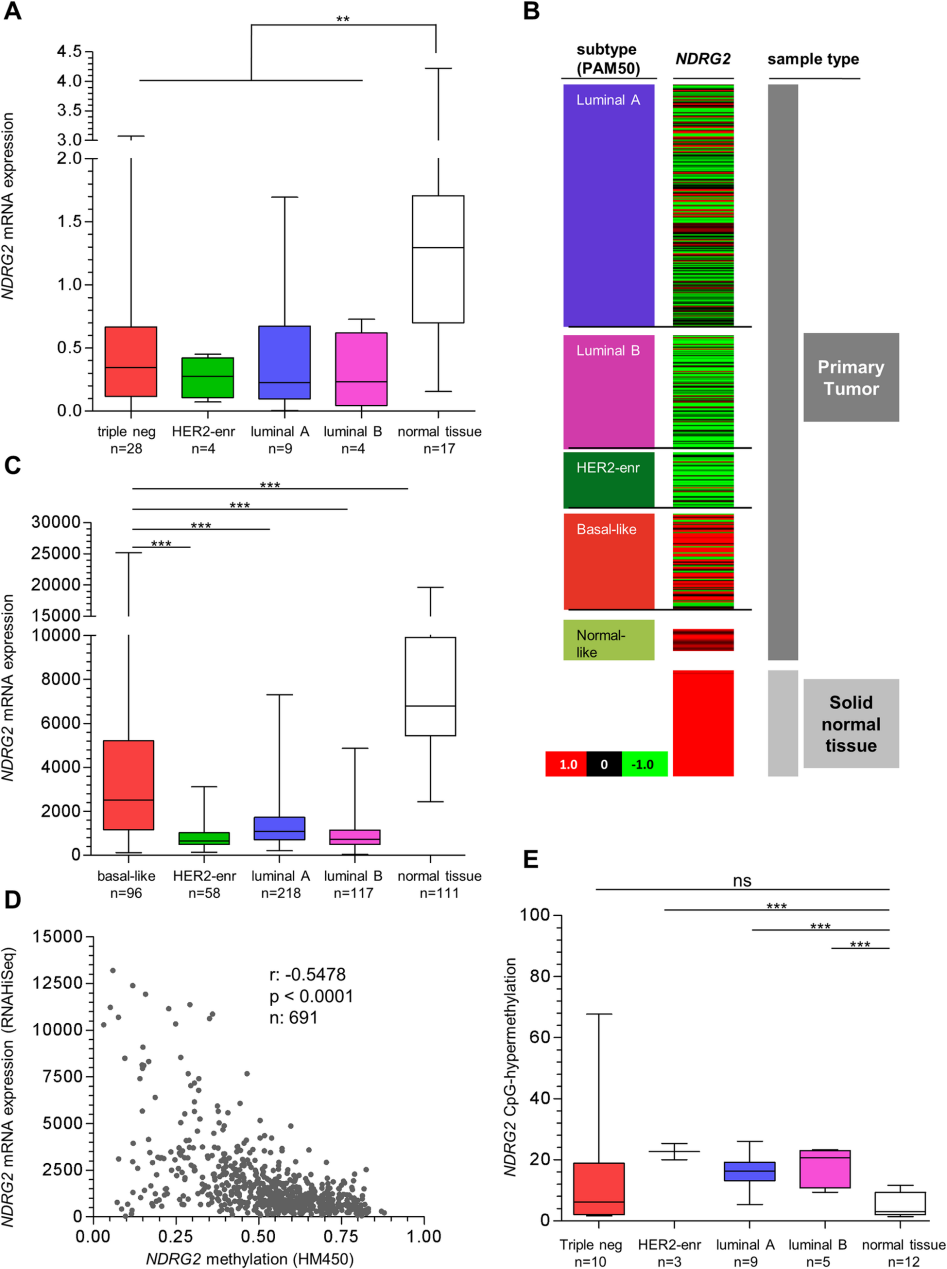
*NDRG2* revealed a divergent expression and methylation pattern in basal- compared to luminal-type breast cancer. **(A)** Box plot analysis (based on our own subtype classified tissue collective) illustrates a decreased *NDRG2* mRNA expression in breast cancer compared with normal breast tissue. An increased *NDRG2* mRNA expression (median expression level: 0.334) in triple negative tumors is observed comparing to HER2-enriched (median expression level: 0.275) and luminal-type (median expression level: 0.229) carcinomas. **(B)** Heatmap of *NDRG2* expression is shown. *Red*: high-, *black*: mean-, *green*: low-expression. *Left panel*: breast cancer subtypes. *Middle panel*: *NDRG2* mRNA expression. *Right panel*: sample type (*dark grey*: primary tumor; *light grey*: solid normal tissues). Breast tumor samples are stratified by subtypes [3]. **(C)** Box plot demonstrating a significant loss of *NDRG2* mRNA expression in luminal-type and HER2-enriched tumors with respect to basal-type cancer specimens. **(D)** Statistical association of *NDRG2* mRNA expression and *NDRG2* hypermethylation in breast cancer. Pearson correlation coefficient: r = -0.5478, P<0.001. **(E)** Box plot analysis (based on our own subtype classified tissue collective) showing significant increased CpG-hypermethylation of the *NDRG2* promoter in luminal-type and HER2-enriched tumors compared to normal tissue samples. Horizontal lines: grouped medians. Boxes: 25–75% quartiles. Vertical lines: range, peak, and minimum. ***P<0.001, **P<0.01, ns: not significant.

Although NDRG2 is thought to be a potential tumor suppressor in breast cancer, tumorigenesis studies investigating the tumor suppressive role of NDRG2 were irrespective of intrinsic breast cancer subtypes or mainly focused on luminal or basal B breast cancer cell models *in vitro* [8,9,15,25] and *in vivo* [12]. To take a deeper look in the biological relevance of NDRG2 regarding the heterogeneous nature of breast cancer, we initially analysed mRNA expression in 45 subtype-classified breast tumors, including triple negative (n = 28), HER2-enriched (n = 4), luminal A (n = 7), luminal B (n = 4) breast cancer specimens, and 15 normal breast tissues by real-time PCR. We showed a significant (P<0.001) loss of *NDRG2* mRNA expression considering all breast cancer subtypes when compared to normal breast tissues (median expression level: 1.3) (Fig 1A). In more detail, we revealed a pronounced downregulation of *NDRG2* mRNA in luminal A- (median FC: 5.7-fold downregulation) and luminal B-type (median FC: 5.6-fold downregulation) breast cancer compared to triple negative (i.e. mammary tumors that lack receptors for estrogen (ER), progesterone (PR), and human epidermal growth factor receptor 2 (HER2)) (median FC: 3.8-fold downregulation) cases (Fig 1A). Next, classifying data of *The Cancer Genome Atlas* (TCGA) by intrinsic breast cancer subtypes based on PAM50 classification [3] we verified an increased downregulation of *NDRG2* mRNA in luminal A- (median FC: 6.2-fold downregulation) and luminal B-type (median FC: 9.4-fold downregulation) breast specimens (Fig 1B and 1C) with respect to normal tissue expression. Again, basal-like (i.e. ER-negative breast tumors characterized e.g. by the expression of cytokeratins 5, 6, 14, and 17 lacking in triple negative cases) tumors more frequently retained abundant *NDRG2* mRNA expression (median FC: 2.7-fold downregulation) (Fig 1B and 1C). In addition to the PAM50 intrinsic subtype classification we showed an association of abundant *NDRG2* expression to ER-, PR-, and HER2-negative breast cancer specimen of the TCGA data cohort (Table 1). Furthermore, we demonstrated a reduced *NDRG2* mRNA expression in invasive ductal carcinoma (IDC) with regard to invasive lobular breast cancer (ILC) (Table 1).

**Table 1:** Clinicopathological parameters in relation to *NDRG2* mRNA expression of the TCGA data portal.

**Variable**	*NDRG2* mRNA expression				
	n^a^	≤ 1067^b^	> 1067^b^	P-value^c^	correlation^d^
**Clinicopathological factors**					
**Age at diagnosis (median: 58 years; range: 26–90 years)**					
≤ 58 years	512	248	264	0.311	-0.032
> 58 years	484	250	234		
**Histological type**					
IDC	727	401	326	**0.001**	**0.102**
ILC	169	43	126		
**Tumor size (pT)**					
pT 1–2	836	430	406	0.061	0.060
pT 3–4	155	67	88		
**Lymph node status (pN)**					
pN negative	473	231	242	0.583	-0.018
pN positive	506	256	250		
**Distant metastasis status (pM)**					
pM negative	845	434	411	0.413	-0.028
pM positive	18	11	7		
**ER status**					
negative	225	82	143	**<0.001**	**-0.144**
positive	722	385	337		
**PR status**					
negative	315	134	181	**0.003**	**-0.095**
positive	629	331	298		
**HER2 status**					
negative	618	322	296	**<0.001**	**-0.146**
positive	103	75	28		

^a^Only female patients with primary and unilateral invasive breast cancer were included

^b^Median *NDRG2* mRNA expression values

^c^Fisher’s exact test at a two-sided significance level of 0.05

^d^Pearson correlation; IDC, invasive ductal carcinoma

ILC, invasive lobular carcinoma; *NDRG2*, N-myc downstream regulated gene 2; ER, Estrogen receptor; PR, Progesterone receptor; HER2, Human epidermal growth factor receptor 2; Significant P-values marked in bold face.

Recent studies investigated promoter hypermethylation as the molecular cause for *NDRG2* expression loss in different cancer types including breast cancer [13]. As expected, by analyzing TCGA data we identified an inverse correlation (Pearson r = -0.548, P<0.001) of *NDRG2* promoter hypermethylation and *NDRG2* mRNA expression in primary breast tumors supporting CpG-hypermethylation as the molecular cause of its gene silencing (Fig 1D). Concerning our own subtype-stratified breast tissue collective we showed a significant (P<0.001) higher CpG-hypermethylation in luminal A (median methylation: 16.3%), luminal B (median methylation: 20.7%) and HER2-enriched (median methylation: 22.7%) tumors with respect to methylation frequency in normal breast tissue. In line with abundant *NDRG2* mRNA expression, CpG-hypermethylation in triple negative tumors (median methylation: 6.2%) was similar to normal breast tissue (median methylation: 3%) (Fig 1E).

### NDRG2 protein is downregulated in the course of breast tumor progression

**Fig 2:**
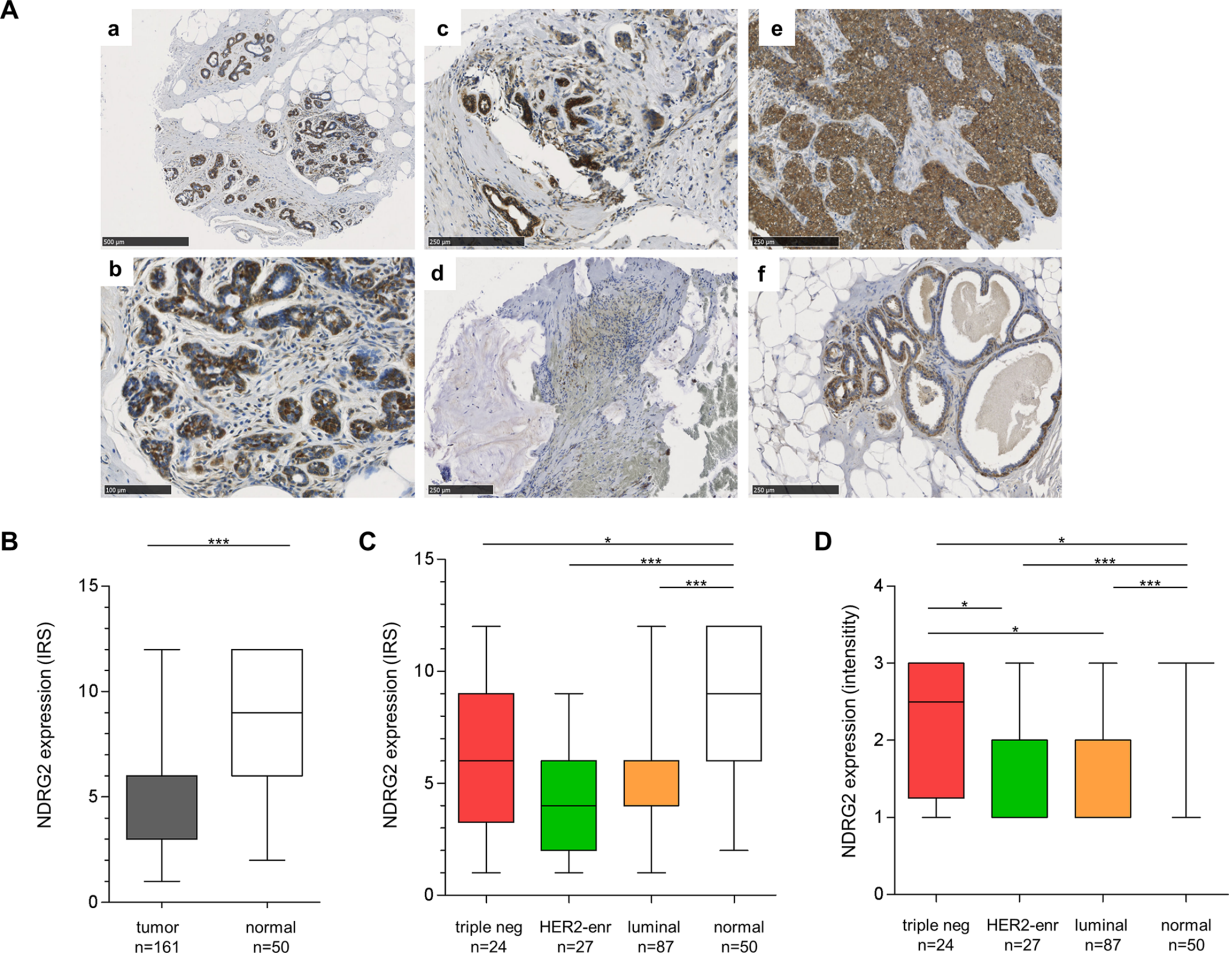
Loss of NDRG2 protein expression in human breast cancer. **(A)** Strong NDRG2 expression in epithelial cells of normal breast tissue (**a** and **b**). Moderate to low NDRG2 immunoreactivity in luminal-type (**c** and **d**) and triple-negative (**e** and **f**) breast carcinoma. **(B)** Box plot analysis showing significant loss of NDRG2 expression in tumor tissue. **(C** to **D)** Box plot analysis illustrating pronounced loss of NDRG2 expression in luminal-type and HER2-enriched tumors (P<0.001) compared to triple negative cancers (P = 0.025) **(C)** and in line with a significant higher median NDRG2 staining intensity in triple negative cancer **(D)**. Horizontal lines: group medians. Boxes: 25–75% quartiles. Vertical lines: range, peak and minimum. ***P<0.001. *P<0.05.

Differential *NDRG2* mRNA expression and CpG-hypermethylation levels between luminal- and basal-like or triple negative breast cancers animated us to investigate NDRG2 protein expression in normal and malignant breast tissue using a TMA containing 161 invasive breast carcinomas and 50 normal breast tissue samples. In general, NDRG2 protein was localized in epithelial cells of the normal breast with absence in fibroblasts, adipocytes and endothelial cells (Fig 2A) with a strong expression (median IRS ≥ 9) in 70% (35/50) of normal breast tissue samples (Fig 2B). However, invasive breast carcinomas showed a significant (P<0.001) reduction or complete loss (median IRS < 6) of NDRG2 expression in 80% (128/161) of cases (Fig 2B). In more detail, stratification of breast cancer specimen in triple negative (n = 24), luminal-type (n = 87) and HER2-enriched (n = 27) breast cancers showed a pronounced downregulation of NDRG2 in luminal-type and HER2-enriched tumors (P<0.001) compared to triple negative cancers (P = 0.025) (Fig 2C). In line, a significant higher intensity of NDRG2 immunohistochemical staining was achieved in triple negative cancers in contrast to luminal-type and HER2-enriched specimens (Fig 2D). Next, clinicopathological characteristics were correlated with NDRG2 immunohistochemistry results for descriptive data analysis. Of interest, strong (IRS ≥ 6) NDRG2 immunohistochemical staining was significantly associated with low HER2-receptor expression (0–1+) concordant to abundant transcriptional *NDRG2* mRNA expression in breast carcinomas with low HER2-expression level (Table 2).

**Table 2:** Correlation of the NDRG2 protein expression with HER2-receptor expression.

	**median NDRG2 protein expression**				
	n^a^	**IRS <6**	**IRS ≥ 6**	correlation^b^	P-value^c^
**HER2 IHC**				**-0.178**	**0.042**
**0–1+**	104	39	65		
**2+– 3+**	27	16	11		

^a^Only female patients with primary and unilateral invasive breast cancer were included

^b^Pearson product-moment correlation coefficient

^c^Fisher’s exact test at a two-sided significance level of 0.05.

IHC, immunohistochemistry; IRS, immunoreactivity score.

### Abundant *NDRG2* mRNA expression in invasive ductal carcinoma is associated with unfavorable survival gene signatures

**Fig 3:**
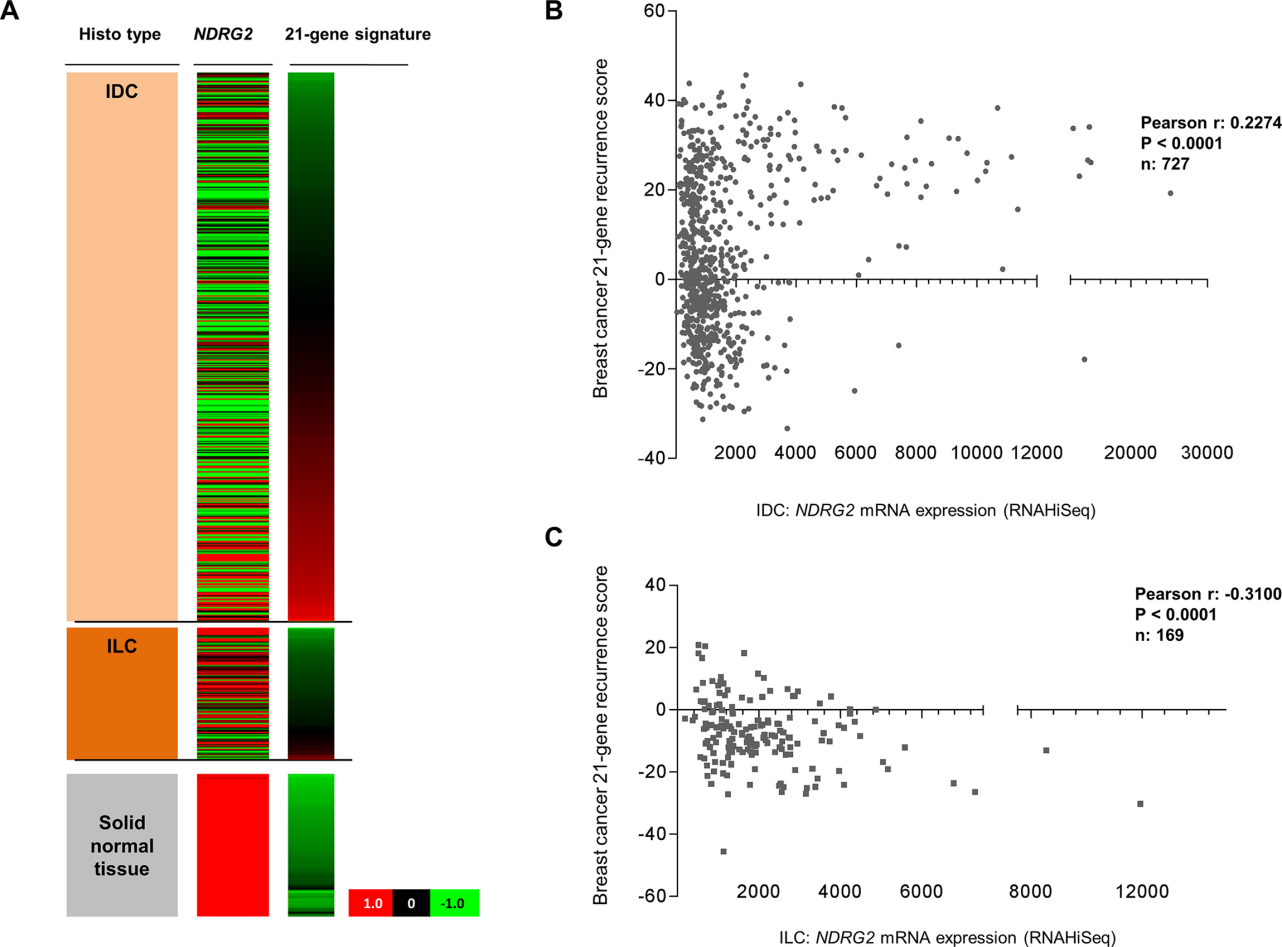
Abundant *NDRG2* mRNA expression in invasive ductal carcinoma is associated with unfavorable patients’ recurrence score. **(A)** Heatmap of *NDRG2* expression and breast cancer 21-gene recurrence score is shown. *Red*: high-, *black*: mean-, *green*: low-expression respectively score values. *Left panel*: breast cancer histological subtypes (*light orange*: invasive ductal carcinoma (IDC); *dark orange*: invasive lobular carcinoma (ILC); *light grey*: solid normal tissues). *Middle panel*: *NDRG2* mRNA expression. *Right panel*: 21-gene signature score values. **(B** to **C)** Statistical association of *NDRG2* mRNA expression and 21-gene recurrence score in **(B)** IDC samples (Pearson correlation coefficient: r = 0.2274, P<0.0001) and **(C)** ILC cases (Pearson correlation coefficient: r = -0.310, P<0.0001).

Based on hormone-receptor and HER2-negative cancer specimen showing abundant NDRG2 expression, we hypothesized that the described NDRG2 expression loss and thus a putative tumor suppressive function of NDRG2 may be confined to luminal-type breast cancers. To address a possible subtype-associated prognostic significance of *NDRG2* mRNA expression we performed a subtype stratified univariate survival analysis on Kaplan Meier-Plotter (KMP) and TCGA data. Analyzing KMP data showed a favorable RFS (P<0.001) and OS (P = 0.014) in luminal A breast cancer patients with abundant NDRG2 mRNA expression underlining the known tumor suppressive function of NDRG2 (S1A and S1B Fig). In contrast, basal-type breast cancer patients showing abundant *NDRG2* expression revealed an unfavorable OS (P = 0.038) and tend to have a worse RFS while significance was barely missed (P = 0.093) (S1C and S1D Fig). Moreover, analyzing distinct gene signatures predicting breast cancer patients’ outcome based on gene expression score values (high vs. low score) [26,27] indicated a clear clinical significance concerning *NDRG2* mRNA expression level (Fig 3). In patients with IDC abundant *NDRG2* mRNA expression showed a significant association (Pearson r: 0.2274, P<0.001) with a high b*reast cancer 21-gene recurrence score* predicting poor prognosis in tamoxifen-treated, node-negative breast cancer [26] (Fig 3A and 3B). Contrary abundant *NDRG2* mRNA expression in patients with ILC is associated with a low recurrence score indicating favorable prognosis (Pearson r: -0.3100, P<0.001) (Fig 3A and 3C). Further, ILC with abundant *NDRG2* expression showed an inverse correlation (Pearson r: -0.3795, P<0.001) with high *breast cancer HRneg/Tneg survival score* associated with poor metastatic outcome in early stage TNBC [27] while correlation in IDC showing abundant NDRG2 was low (Pearson r: -0.0736, P = 0.0474) (S2 Fig). With respect to hormone-receptor positive breast cancer (i.e. particularly histological invasive lobular and intrinsic luminal breast carcinoma) these data underline a tumor suppressive role of *NDRG2* probably depending on positive hormone-receptor expression.

### NDRG2 loss-of-function and gain-of-function *in vitro* tumor models in basal B and luminal-type breast cancer cells

To provide for the first time insight into NDRG2 biology beyond the assumed tumor suppressive role in luminal- and basal B-type breast carcinoma cell models, we established two differential basal A-type *in vitro* tumor models: (I) A small-interfering RNA (siRNA)-mediated NDRG2 knockdown loss-of-function model in HCC1806 breast cancer cells showing abundant wild type (WT) NDRG2 expression and (II) NDRG2 over-expression in a gain-of-function model using a full-length NDRG2-pT-Rex-DEST 30 expression vector (+NDRG2) or the empty vector alone (-NDRG2) in WT BT20 tumor cells showing no endogenous NDRG2 expression. To address published tumor suppressive function of NDRG2 in luminal-type breast cancer we performed NDRG2 over-expression in luminal MCF7 cells showing low endogenous NDRG2 expression.

**Fig 4:**
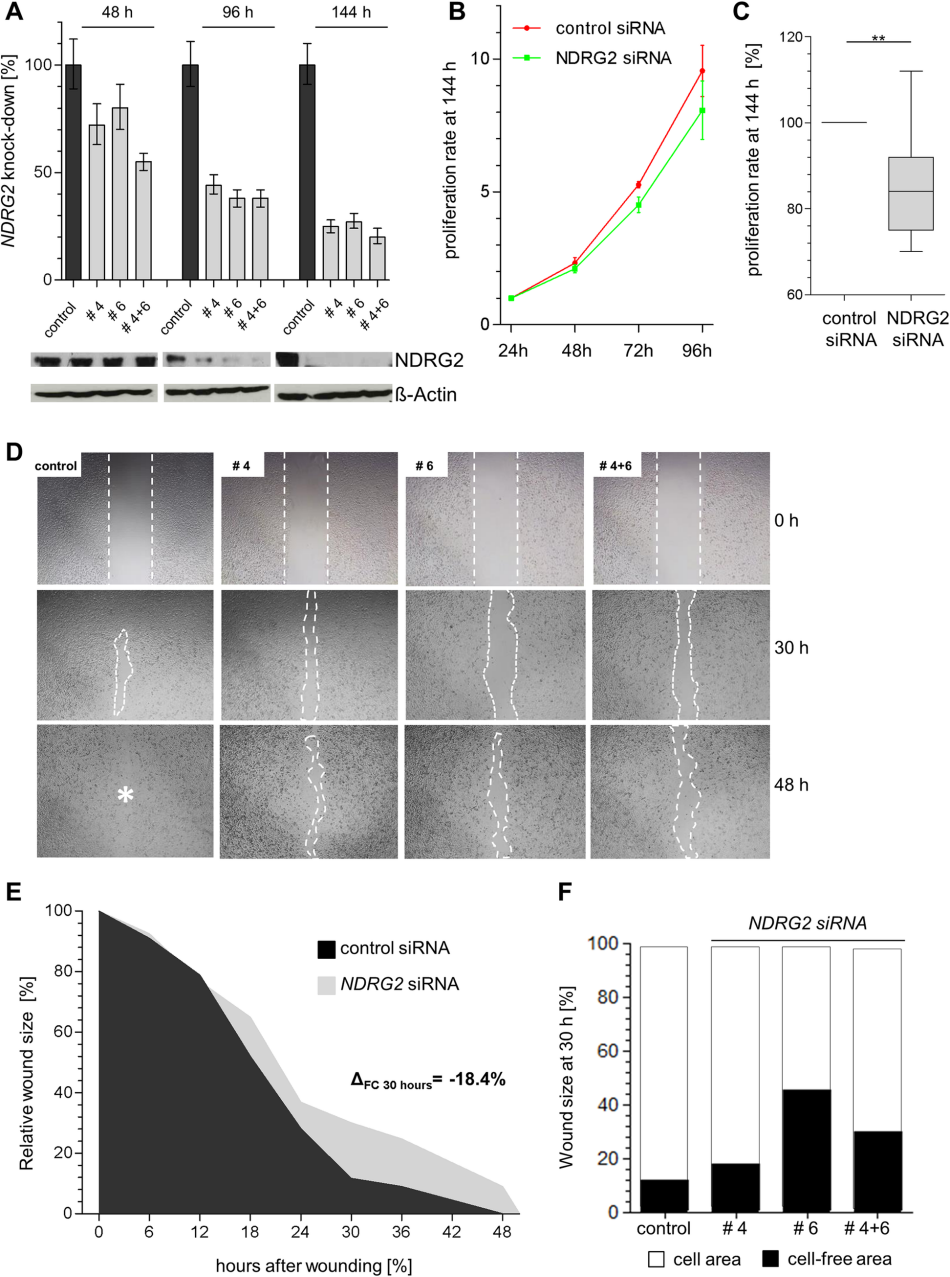
NDRG2 expression loss leads to reduced cell proliferation and migration in basal type HCC1806 cells. **(A)** NDRG2 knockdown in basal-type HCC1806 cells. *Upper graph*: *NDRG2* mRNA expression loss after 48 h, 96 h and 144 h RNA interference treatment. *Vertical lines*: standard deviation of three independent analyses. House-keeping gene *GAPDH* was used for normalization. *Lower graph*: Representative western blot illustrates the NDRG2 protein expression loss after 48 h, 96 h and 144 h RNA interference treatment. β-Actin served as loading control. **(B** to **C)** Cell proliferation due to transient NDRG2 knockdown: **(B)** Cell proliferation rate is decreased due to NDRG2 expression loss (green line; median proliferation rate of NDRG2-specific siRNA #4, #6 as well as a combination of #4 and #6) compared to HCC1806 cells treated with control siRNA (red line). *Vertical lines*: *standard error of mean* (SEM) of triplicates **(C)** Box plot represents averages of triplicate experiments. (**D** to **F**) Cell migration was analyzed by performing a scratch wound healing assay. **(D)** Representative images of the wound size are shown for HCC1806-siRNA control and HCC1806-NDRG2 siRNA for 0 h, 30 h and 48 h is shown. **(E)** Mean migration rate of a control cell set (HCC1806-siRNA control) and independent HCC1806-NDRG2 siRNA cells over 48 h is shown. Cell-free area on day 0 was set as 100% and used for standardization. Δ_FC 30 hours_: differences of cell-free areas after 30 h. **(F)** Detailed comparison of wound closure after 30 h.

Two high quality and independent commercial siRNAs (#4 and #6) and a combination of both were used for all transient knockdown experiments. Using *NDRG2*-specific siRNAs a complete loss of NDRG2 protein was achieved after a 144 h treatment (Fig 4A), a knockdown of the respective mRNA of 76% (#4), 73% (#6) and 80% (combination of #4 and #6) was demonstrated (Fig 4A). For all following experiments, cells were treated with siRNAs for 144 h to assure an efficient NDRG2 protein knockdown. In contrast, the negative control, i.e. a non-coding (nc)-siRNA, exhibited *NDRG2* expression similar to the HCC1806 WT, which indicated the absence of unspecific side effects potentially caused by transfection procedures. On the other hand, BT20 and MCF7 cells transient transfected with full-length NDRG2-pT-Rex-DEST 30 expression vector showed a median expression fold change (FC) of 161 and 147 compared to NDRG2-negative cells, respectively (S3A and S3B Fig).

### Abundant NDRG2 expression forces proliferation and migration in basal A-type HCC1806 and BT20 cells

Addressing the potentially biological role of NDRG2 in basal-type breast cancer carcinogenesis, the impact of NDRG2 expression on tumor cell proliferation and migration was studied based on our *in vitro* tumor models. In HCC1806 cells proliferation was significantly (P = 0.007) reduced by siRNA knockdown of NDRG2 compared to cells treated with control siRNA (Fig 4B and 4C). Of interest, in BT20 cells, we observed that in a bulk of transiently transfected cells, i.e., a part of non-transfected BT20 cells may be present, NDRG2 re-expression led to a significant (P = 0.042) increased cell growth by 38%, compared with the mock-transfected cells 96 h after plating (S3C Fig). However, measurement of cell proliferation by using XTT reagent revealed no significant influence of NDRG2 over-expression on BT20 cell proliferation (S3E Fig). Underlining the published functional consequences of NDRG2 re-expression in luminal type breast cancer we demonstrated a 26% decreased proliferation rate in NDRG2 over-expressing MCF7 compared to control cells while significance was barely missed (P = 0.064) (S3F Fig).

Next, we focused on cell migration performing a wound healing assay. While the motility of BT20 cells was not clearly altered by NDRG2 overexpression (data not shown), loss of NDRG2 expression inhibits cell migration of basal-type HCC1806 cells, i.e. HCC1806 NDRG2 positive cells repopulated the wounded area notably faster than corresponding NDRG2 negative cells (Fig 4D–4F): After 48 h HCC1806-control siRNA cells repopulated the wounded area completely compared to corresponding HCC1806 cells treated with NDRG2 siRNA (Fig 4D and 4E). In more detail, 30 h after wounding, cell movement of HCC1806-NDRG2 siRNA cells was maximal reduced (Fig 4E). At this time point HCC1806-control cells had repopulated 88.3% of the wound, whereas HCC1806-NDRG2 siRNA cells covered on average 70% of the scratch area. A detailed comparison of wound closure between different NDRG2 and negative siRNAs 30 h after wounding is shown in Fig 4F.

## Discussion

Today, several lines of evidence suggest a potential suppressive role of NDRG2 in tumorigenesis. Current studies in colon [6] and breast carcinoma [12] revealed that NDRG2 antagonizes transforming growth factor β (TGF-β)–mediated cell invasion. In addition, it has been demonstrated that NDRG2 inhibits tumor cell proliferation and increases p53- or hypoxia-mediated apoptosis and its expression is correlated with patient survival and prognosis [4,8,9,28–30]. In a recent study, Ma *et al*. [31] demonstrated an association of abundant NDRG2 expression with glucose transport in breast carcinoma cells associated with a favorable patients’ outcome. However, studies evaluating the putative tumor suppressive biological and clinical impact of NDRG2 in breast cancer were irrespective of intrinsic breast cancer subtypes or as mentioned in this study before, mainly based on luminal- and basal B-type cell models. The current study is the first to analyze in depth NDRG2 expression, as well as its potential clinical and functional impact toward intrinsic breast cancer subtypes.

Initially, we verified by both real-time PCR and immunohistochemistry that NDRG2 was downregulated in human breast tumor tissue, underlining recent studies showing NDRG2 expression loss in the course of tumor progression. We correlated NDRG2 loss to the breast cancer subtypes as defined by St. Gallen criteria [18] and PAM50 [3] in two independent tissue collectives. The St. Gallen criteria characterised breast tumors mainly on the expression level of ER, PR and HER2, defining triple negative breast cancer (TNBC) by the lack of these receptors. In contrast, the PAM50 array investigates expression of distinct gene signatures (including a basal-like gene expression signature) for classification of breast tumors. Therewith, the majority of TNBC (∼ 70%) falls into the classification of the basal-like subtype characterized e.g. by the expression of cytokeratins 5, 6, 14, and 17 [32,33]. Interestingly, *NDRG2* downregulation was abundantly found in luminal A, luminal B and HER2-enriched breast cancer while TNBC (own tissue collective, St. Gallen criteria) and basal-like tumors (TCGA data, PAM50) more frequently retained *NDRG2* mRNA expression. Next, to analyze the molecular cause of downregulation, we investigated the epigenetic configuration of the *NDRG2* gene promoter in our own subtype-stratified breast tissue collective, as it is known that the *NDRG2* promoter sequence contains distinct CpG islands. In fact, *NDRG2* gene promoter was methylated in 66% of the analyzed breast tumor tissues, while in TNBC median methylation was similar to normal breast tissue methylation. Again, TCGA data analyses confirmed our results by indicating a frequent hypermethylation in primary tumor tissue and, accordingly, demonstrated an inverse correlation (r = –0.548, P<0.001) of *NDRG2* methylation and mRNA expression indicating promoter hypermethylation as the molecular cause of the *NDRG2* loss particularly in luminal and HER2-enriched breast cancer.

Previous studies indicated that the expression of NDRG2 is regulated by many hormones, including dexamethasone, insulin, androgens, and aldosterone [34–36]. In addition, analysis of the promoter region flanking 5’ of the *NDRG2* gene revealed a putative estrogen-response element (ERE), which suggests that estrogen may also play a regulative role of NDRG2 expression [37]. Furthermore, a recent study by Ma *et al*. [38] showed a co-localization of NDRG2 with the estrogen receptor β (ERβ) in astrocytes and up-regulation of NDRG2 expression by estrogen. In agreement with a possible hormone-depending regulation of NDRG2 expression, we demonstrated a positive association of abundant *NDRG2* mRNA expression with ILC which is almost always hormonally regulated (90–95% of cases express ERα and ERβ) [39]. Owing to that we further revealed a significant correlation of abundant *NDRG2* mRNA expression in ILC concerning a defined low recurrence [26] and metastasis prediction [27] score. Unexpectedly, abundant *NDRG2* expression in IDC significantly associated with a high recurrence and metastasis prediction score, i.e. unfavorable patients’ outcome. Moreover, the described potential clinical tumor suppressive impact of abundant *NDRG2* mRNA expression is impaired in hormone-receptor negative breast cancer: While in luminal A breast cancer patients, therewith ER-positive tumors, abundant *NDRG2* expression significantly predicted both a favorable RFS and OS, patients with basal-like breast cancer showed worse prognosis upon increased *NDRG2* mRNA expression. Of clinical importance, in breast cancer that may be or is hormone-receptor negative, like invasive ductal and basal-like cancer, transcriptional regulation of the *NDRG2* gene seems to be irrespective of hormone expression thus the putative tumor suppressive function of NDRG2 may be confined to luminal-type breast cancers. Besides the putative estrogen-dependent regulation of NDRG2 in luminal type breast cancer, divergent expression profile of NDRG2 may be due to the metastatic behaviour of basal-like tumors. In this context, Smid et al. [40] found a 648-gene signature (including *NDRG2)* up-regulated in basal type breast cancer. Underlining the reversal prognostic impact of *NDRG2* in basal- compared to luminal-type breast cancer, the tumor suppressor gene *SFRP1*, a key antagonist of the WNT/β-catenin signaling pathway, also tends to be associated with unfavorable patients’ outcome (Kaplan Meier-Plotter data) in basal-like breast cancer. Since basal-type tumors frequently metastasize to the brain, one may speculate that high expression of NDRG2 as well as WNT/β-catenin signaling molecules, known to have an important putative role in the development and maintenance of normal brain tissue [41–44], facilitate metastasis of basal tumors. Thus NDRG2 could support the thesis that the seed grows better in the soil it resemble [45] as mentioned by Smid et al. Since biological evidence supporting this hypothesis was lacking, the present study aimed to proof the relevance of NDRG2 in basal-type breast cancer in two independent transient basal A-type *in vitro* cell models: BT20 and HCC1806 belong to the basal A subtype while published MDA-MB-231 cell models, showing a tumor suppressive function of NDRG2, belongs to the basal B subtype. Stratification of basal type breast cancer cell lines into two subgroups was firstly demonstrated 2006 by Neve et al. [46] and could be validated by further studies [47–49]. In general, basal B cell lines were characterized by markers associated with aggressive tumor features including those involved in epithelial-mesenchymal transition (EMT) [48,49]. In more detail, basal B cells reveal positive expression of the mesenchymal-specific cell protein vimentin and exhibit a stem-cell like expression profile. Therewith, basal B cells like e.g. MDA-MB-231 are more accurately classified as a mesenchymal or mesenchymal stem like triple negative breast cancer (TNBC) cell line reflecting the clinical “triple-negative” tumor type rather than basal-like tumors [46,48,49]. In contrast, basal A cells were characterized by the expression of basal cytokeratins 5, 6, 14, and 17 and lack of vimentin expression as clear evidence for basal origin [46,48,49]. Of interest, comparison of expression patterns between subtype classified cell lines and 86 breast tumors by Kao et al. [48] showed all basal-like tumors most resembled basal A cancer cell lines. Since the basal A lines cluster matches closely the PAM50 gene expression signature [46] we performed functional NDRG2-analysis in basal A lines to shed light on the basal-like subtype beyond the impact of NDRG2 mainly shown in mesenchymal basal B cell lines. In parallel to the abundant NDRG2 expression in basal-type primary breast cancer, we observed a clear tumor suppressive impact mediated by NDRG2 knockdown in metastatic, basal A-like HCC1806. In fact, cell proliferation in HCC1806 cells was effectively suppressed by NDRG2 knockdown. Consistent with that, a wound healing assay confirmed an inhibition of cell motility of metastatic HCC1806 cells upon NDRG2 expression loss. In addition to that, we revealed an increased cell growth upon NDRG2 over-expression in BT20 cells lacking endogenous NDRG2 and demonstrated a 26% decreased proliferation rate in NDRG2 over-expressing luminal-type MCF7.

In summary, we provide for the first time clinical and functional evidence that the described putative tumor suppressive function of NDRG2 may be confined to luminal-type and basal B-type (more reflecting mesenchymal TNBC) breast cancers. Our data propose a fundamental clinical tumor suppressive role of NDRG2 in hormone-receptor positive breast cancer while in basal-like breast cancer patient’s abundant NDRG2 expression is associated with unfavorable patients’ outcome and a more aggressive phenotype *in vitro*. Further investigations considering transcriptional *NDRG2* regulation and clinical significance in basal-type cancer are needed that may help to understand underlying pathways in more detail, finally helping to improve disease management.

## Supporting Information

S1 Fig NDRG2 expression in human basal-type breast tumors predicts unfavorable overall (OS) and recurrence-free (RFS) survival in an independent data set. (**A** to **B**) Kaplan-Meier analyses illustrating RFS **(A)** and OS **(B)** of luminal A-type breast cancer patients with high *NDRG2* (red curve) compared to reduced *NDRG2* expression (black curve). **(C** to **D)** Survival curves display RFS (**C**) and OS (**D**) of basal-type breast cancer patients with high *NDRG2* (red curve) compared to reduced *NDRG2* expression (black curve).
(TIF)

S2 Fig Abundant *NDRG2* mRNA expression in invasive ductal carcinoma is associated with unfavorable patients’ recurrence score. **(A)** Heatmap of *NDRG2* expression and breast cancer triple negative (TNBC) score is shown. *Red*: high-, *black*: mean-, *green*: low-expression respectively score values. *Left panel*: breast cancer histological subtypes (*light orange*: invasive ductal carcinoma (IDC); *dark orange*: invasive lobular carcinoma (ILC); *light grey*: solid normal tissues). *Middle panel*: *NDRG2* mRNA expression. *Right panel*: TNBC-gene signature score values. **(B** to **C)** Statistical association of *NDRG2* mRNA expression and TNBC-gene signature score in **(B)** IDC samples (Pearson correlation coefficient: r = 0.2274, P<0.0001) and **(C)** ILC cases (Pearson correlation coefficient: r = -0.310, P<0.0001).
(TIF)

S3 Fig Forced NDRG2 expression promotes cell proliferation in basal-type BT20 cells and decreases cell proliferation in luminal-type MCF7 cells. NDRG2 expression in basal-type BT20 **(A)** and luminal-type MCF7 **(B)**. *Upper graph*: *NDRG2* mRNA expression after transiently transfection. *Vertical lines*: standard deviation of three independent analyses. *GAPDH* expression was used for normalization. *Lower graph*: Representative western blot illustrating NDRG2 protein expression after transient transfection. β-Actin served as loading control. **(C** to **F)** Cell number is increased in BT20 cells following NDRG2 over-expression **(C** and **E)** or decreased in MCF7 cells **(D** and **F)**. *Vertical lines*: standard error of mean (SEM) of three independent experiments.
(TIF)

S1 Table Clinicopathological breast cancer patients’ data of the TCGA portal. (DOCX)

S2 Table Clinicopathological data of the subtype-specific patients’ tissue collective. (DOCX)

S3 Table Sequences for the real-time PCR and pyrosequencing primer and performing conditions. (DOCX)
