# Supplementary figures and images for: Abundant NDRG2 Expression Is Associated with Aggressiveness and Unfavorable Patients’ Outcome in Basal-Like Breast Cancer
Source: PLoS One. 2016 Jul 11;11(7):e0159073. doi: 10.1371/journal.pone.0159073 (PMC4939972; doi:10.1371/journal.pone.0159073)

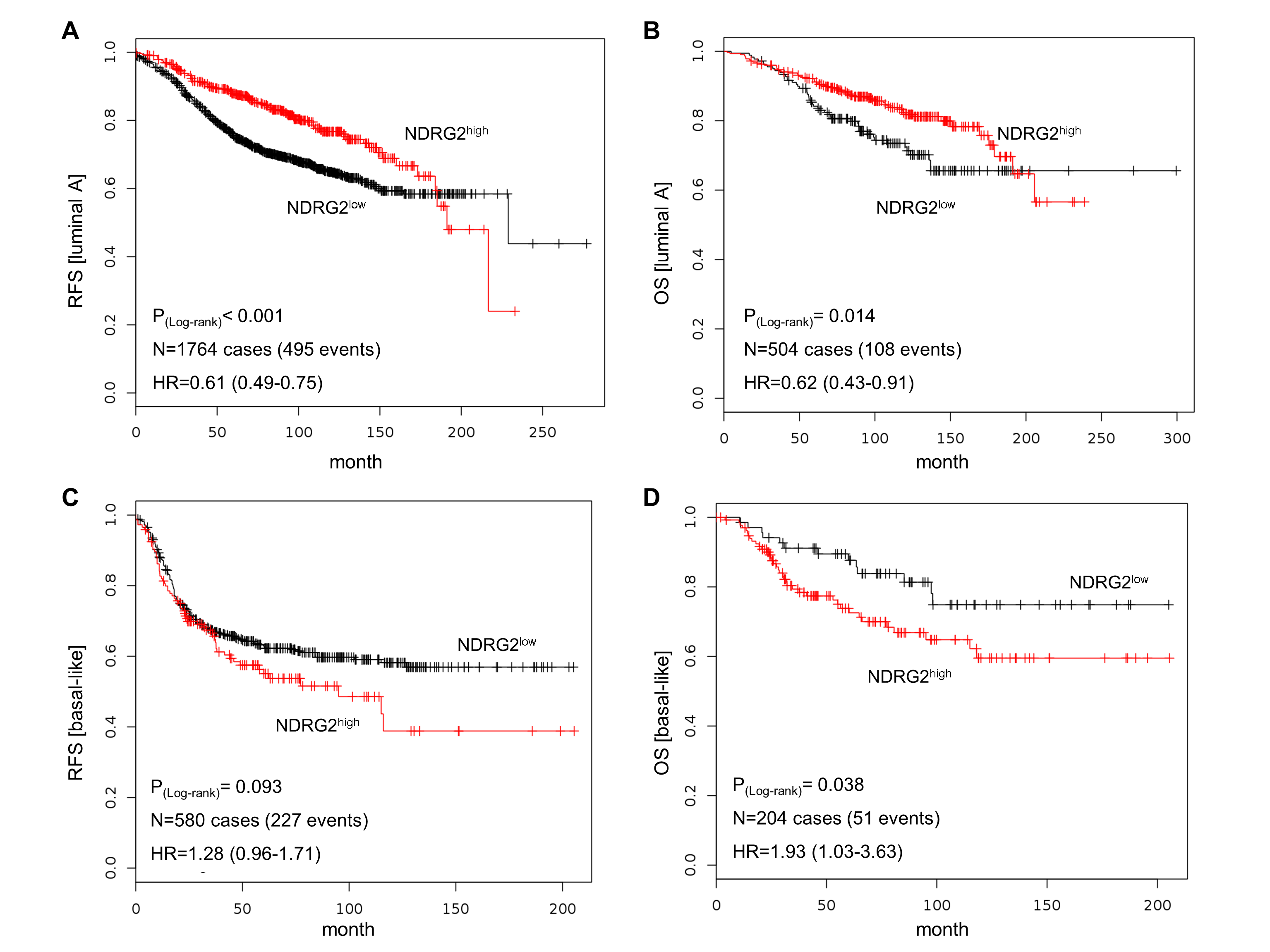

Supplement: S1 Fig — (A to B) Kaplan-Meier analyses illustrating RFS (A) and OS (B) of luminal A-type breast cancer patients with high NDRG2 (red curve) compared to reduced NDRG2 expression (black curve). (C to D) Survival curves display RFS (C) and OS (D) of basal-type breast cancer patients with high NDRG2 (red curve) compared to reduced NDRG2 expression (black curve). (TIF) [file pone.0159073.s001.tif]

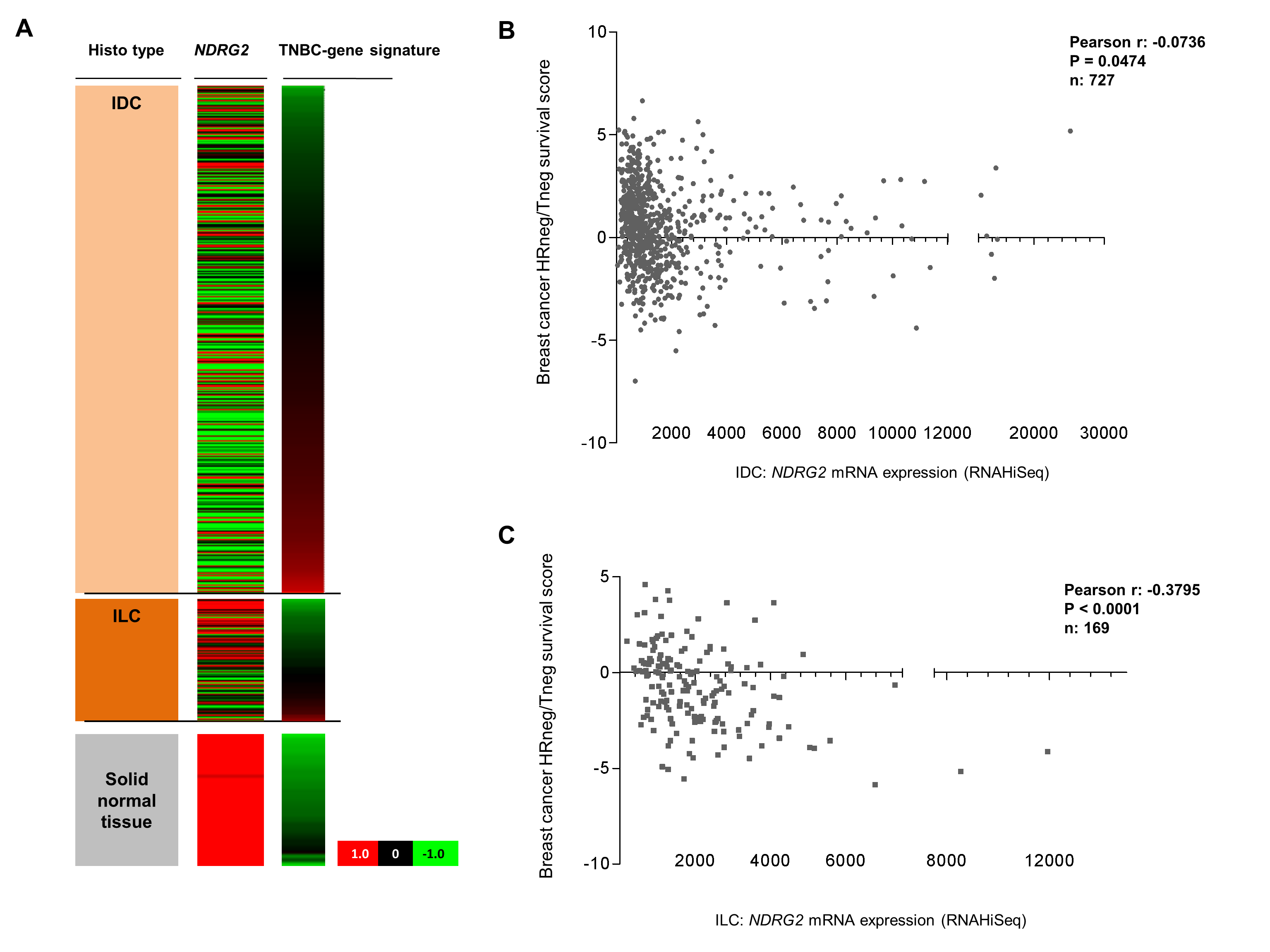

Supplement: S2 Fig — (A) Heatmap of NDRG2 expression and breast cancer triple negative (TNBC) score is shown. Red: high-, black: mean-, green: low-expression respectively score values. Left panel: breast cancer histological subtypes (light orange: invasive ductal carcinoma (IDC); dark orange: invasive lobular carcinoma (ILC); light grey: solid normal tissues). Middle panel: NDRG2 mRNA expression. Right panel: TNBC-gene signature score values. (B to C) Statistical association of NDRG2 mRNA expression and TNBC-gene signature score in (B) IDC samples (Pearson correlation coefficient: r = 0.2274, P<0.0001) and (C) ILC cases (Pearson correlation coefficient: r = -0.310, P<0.0001). (TIF) [file pone.0159073.s002.tif]

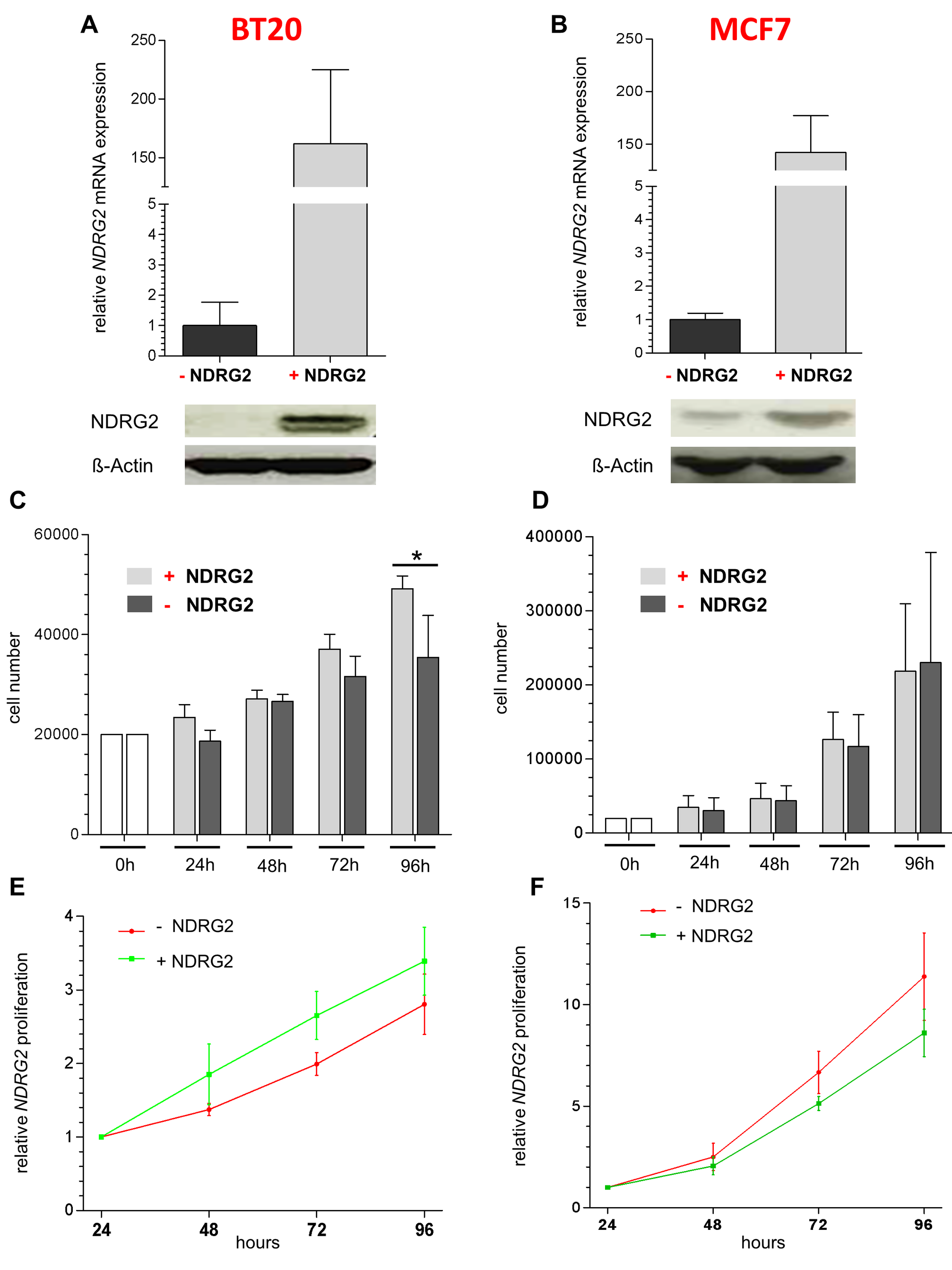

Supplement: S3 Fig — NDRG2 expression in basal-type BT20 (A) and luminal-type MCF7 (B). Upper graph: NDRG2 mRNA expression after transiently transfection. Vertical lines: standard deviation of three independent analyses. GAPDH expression was used for normalization. Lower graph: Representative western blot illustrating NDRG2 protein expression after transient transfection. β-Actin served as loading control. (C to F) Cell number is increased in BT20 cells following NDRG2 over-expression (C and E) or decreased in MCF7 cells (D and F). Vertical lines: standard error of mean (SEM) of three independent experiments. (TIF) [file pone.0159073.s003.tif]
