# Supplementary material for: Abundant NDRG2 Expression Is Associated with Aggressiveness and Unfavorable Patients’ Outcome in Basal-Like Breast Cancer
Source: PLoS One. 2016 Jul 11;11(7):e0159073. doi: 10.1371/journal.pone.0159073 (PMC4939972; doi:10.1371/journal.pone.0159073)
Supplement: S1 Table — (DOCX) [file pone.0159073.s004.docx]

**S1 Table. Clinicopathological breast cancer patients’ data of the TCGA portal**

| **Variable** | **n^a^** | **%** | |
| --- | --- | --- | --- |
|  |  |  |  |
| **Age at diagnosis (median: 58 years)** |  |  |  |
| ≤ 58 years | 514 |  | 51.5 |
| > 58 years | 485 |  | 48.5 |
| **Histological type** |  |  |  |
| IDC | 730 |  | 73.1 |
| ILC | 169 |  | 16.9 |
| Other | 100 |  | 10.0 |
| **Tumor size (pT)** |  |  |  |
| pT 1–2 | 839 |  | 84.0 |
| pT 3–4 | 155 |  | 15.5 |
| Unknown | 5 |  | 5.0 |
| **Lymph node status (pN)** |  |  |  |
| pN negative | 474 |  | 47.4 |
| pN positive | 508 |  | 50.9 |
| Unknown | 17 |  | 1.7 |
| **Distant metastasis status (pM)** |  |  |  |
| pM negative | 848 |  | 84.9 |
| pM positive | 18 |  | 1.8 |
| Unknown | 133 |  | 13.3 |
| **ER status** |  |  |  |
| negative | 226 |  | 22.6 |
| positive | 724 |  | 72.5 |
| Unknown | 49 |  | 4.9 |
| **PR status** |  |  |  |
| negative | 318 |  | 31.8 |
| positive | 629 |  | 63.0 |
| Unknown | 52 |  | 5.2 |
| **HER2 status** |  |  |  |
| negative | 621 |  | 62.2 |
| positive | 103 |  | 10.3 |
| Unknown | 275 |  | 27.5 |

^a^Only female patients with primary, unilateral, invasive breast cancer were included; IDC, invasive ductal carcinoma; ILC, invasive lobular carcinoma; ER, Estrogen receptor; PR, Progesterone receptor; HER2, Human epidermal growth factor receptor 2.
