# Supplementary material for: Abundant NDRG2 Expression Is Associated with Aggressiveness and Unfavorable Patients’ Outcome in Basal-Like Breast Cancer
Source: PLoS One. 2016 Jul 11;11(7):e0159073. doi: 10.1371/journal.pone.0159073 (PMC4939972; doi:10.1371/journal.pone.0159073)
Supplement: S2 Table — (DOCX) [file pone.0159073.s005.docx]

**S2 Table. Clinicopathological data of the subtype-specific patients’ tissue collective.**

| **Clinicopathological factors** | **n^a^** | **%** |
| --- | --- | --- |
| **Age at diagnosis (median: 59 years; range 33-82 years)** |  |  |
| ≤ 59 years | 21 | 47.7 |
| > 59 years | 20 | 45.5 |
| Unknown | 3 | 6.8 |
| **Tumor size (pT)** | | |
| pT 1 | 12 | 27.3 |
| pT 2 | 21 | 47.7 |
| pT 3 | 5 | 11.4 |
| pT 4 | / | 7 |
| Unknown | 6 | 13.6 |
| **Grade** | | |
| G1 | / | / |
| G2 | 10 | 22.7 |
| G3 | 30 | 68.2 |
| Unknown | 4 | 9 |
| **Lymph node status (pN)** | | |
| pN negative | 19 | 43.2 |
| pN positive | 18 | 43.2 |
| Unknown | 6 | 13.6 |
| **ER status** | | |
| Negative (0-2) | 27 | 61.4 |
| Positive (3-12) | 12 | 27.3 |
| Unknown | 5 | 11.4 |
| **PR status** | | |
| Negative (0-2) | 27 | 61.4 |
| Positive (3-12) | 12 | 27.3 |
| Unknown | 5 | 11.4 |
| **HER2 status** | | |
| Negative (0 – 2+) | 37 | 84.1 |
| Positive (3+) | 5 | 11.4 |
| Unknown | 2 | 4.5 |

^a^Only female patients with primary, unilateral, invasive breast cancer were included; IDC, invasive ductal carcinoma; ILC, invasive lobular carcinoma; ER, Estrogen receptor; PR, Progesterone receptor; HER2, Human epidermal growth factor receptor 2.
