# Supplementary material for: Abundant NDRG2 Expression Is Associated with Aggressiveness and Unfavorable Patients’ Outcome in Basal-Like Breast Cancer
Source: PLoS One. 2016 Jul 11;11(7):e0159073. doi: 10.1371/journal.pone.0159073 (PMC4939972; doi:10.1371/journal.pone.0159073)
Supplement: S3 Table — (DOCX) [file pone.0159073.s006.docx]

**S3 Table. Sequences for the real-time PCR and pyrosequencing primer and performing conditions**

| **Gene** | **Sequence (5' → 3')** | **T_A_** | **Cycles** | **Product size** |
| --- | --- | --- | --- | --- |
| ***NDRG2* F** | TGGGACTCAACTATAAATCTTGCTT | 60°C | 40 | 146 bp |
| ***NDRG2* R** | TGGGTACTGATATCCCAAAGG |  |  |  |
|  |  |  |  |  |
| ***NDRG2* F** | GAGAGGGAYGAGGTAGATTTTGAGATTAT | 56°C | 45 | 227 bp |
| ***NDRG2* R-bio** | Biotin-AAACCCCCAAAAACTCTAACTCCTAAAT |  |  |  |
| ***NDRG2* S** | GTTTTTTTTAAGTTTTTATTTTATTTT |  |  |  |

F, forward; R, reverse; S, sequencing, bio, biotinylated; T_A_, annealing temperature
